# Complex Trait Loci in Maize Enabled by CRISPR-Cas9 Mediated Gene Insertion

**DOI:** 10.3389/fpls.2020.00535

**Published:** 2020-05-05

**Authors:** Huirong Gao, Jasdeep Mutti, Joshua K. Young, Meizhu Yang, Megan Schroder, Brian Lenderts, Lijuan Wang, Dave Peterson, Grace St. Clair, Spencer Jones, Lanie Feigenbutz, Wally Marsh, Min Zeng, Susan Wagner, Jeffry Farrell, Kay Snopek, Chris Scelonge, Xiaoyi Sopko, Jeffry D. Sander, Scott Betts, A. Mark Cigan, N. Doane Chilcoat

**Affiliations:** Research and Development, Corteva Agriscience, Johnston, IA, United States

**Keywords:** maize, CRISPR-Cas9, complex trait loci, trait stack, gene target, gene expression, recombinase-mediated cassette exchange, genetic crossing

## Abstract

Modern maize hybrids often contain biotech and native traits. To-date all biotech traits have been randomly inserted in the genome. Consequently, developing hybrids with multiple traits is expensive, time-consuming, and complex. Here we report using CRISPR-Cas9 to generate a complex trait locus (CTL) to facilitate trait stacking. A CTL consists of multiple preselected sites positioned within a small well-characterized chromosomal region where trait genes are inserted. We generated individual lines, each carrying a site-specific insertion landing pad (SSILP) that was targeted to a preselected site and capable of efficiently receiving a transgene via recombinase-mediated cassette exchange. The selected sites supported consistent transgene expression and the SSILP insertion had no effect on grain yield. We demonstrated that two traits residing at different sites within a CTL can be combined via genetic recombination. CTL technology is a major step forward in the development of multi-trait maize hybrids.

## Introduction

In the early years of biotech crop cultivation, a single transgene was used to confer resistance to insects or tolerance to herbicides. This benefited growers by reducing yield losses from pests and weeds, while at the same time reducing soil erosion and the use of chemical pesticides (Koziel et al., 1993; Padgette et al., 1995). Over the last ∼30 years some insects have developed resistance to insecticidal proteins used in first generation products and herbicide-resistant weeds have become problematic. The need for new traits that are durable and broad-spectrum has been partially met by the introduction of products that employ multiple transgenes (Que et al., 2010). Additionally, the objectives of transgenic traits have expanded, with research in many areas including disease resistance, drought tolerance, nitrogen use efficiency, and grain quality. Traditionally, multiple traits have been brought together using genetic backcrossing, referred to as trait introgression (TI) (Peng et al., 2014). However, introgression of more than four traits which reside at different part of genome in an inbred line is impractical in developing commercial products (Mumm and Walters, 2001; Petolino and Kumar, 2016; Chen and Ow, 2017). Moreover, backcrossing introgression brings along unintended genome sequences adjacent to the transgene which can lower yield. To overcome these challenges, improved methods for trait assembly are needed.

Several approaches have been taken to improve trait assembly. TI has been enhanced using molecular markers which facilitate selection and reduce the number of backcrosses needed (Peng et al., 2014). Transgenic constructs containing multiple gene expression cassettes have been used, but this approach is inefficient (Cao et al., 2002; Dafny-Yelin and Tzfira, 2007; Que et al., 2010). Sequential transformation methods have been shown to enable stacking traits by inserting a transgene immediately adjacent to an existing biotech trait using recombinase-mediated cassette exchange (RMCE) or zinc finger/homing endonucleases (Ow, 2011; Ainley et al., 2013; D’Halluin et al., 2013; Kumar et al., 2015; Petolino and Kumar, 2016; Srivastava and Thomson, 2016). Although modular trait stacking resolved some of the issues associated with the direct transformation of a large plasmid, it has not been adopted for product development in part due to concern that the newly added transgene may alter expression of the original transgene because of spatial proximity and partly because the process has low efficiency. Both of those molecular stacking approaches are inflexible; should one of the trait genes not be required in a geography or have lost efficacy, it can’t be easily separated or replaced by conventional breeding.

To facilitate development of multi-trait products in maize, we have developed a complex trait locus (CTL) approach. A CTL is comprised of multiple gene-targeting sites carefully positioned within a small well-characterized region in the genome. To create a CTL, individual transgenic lines are created that have a site-specific insertion landing pad (SSILP) targeted to a preselected site using the CRISPR-Cas9 system. Each of the SSILP lines are capable of receiving a transgene via high-efficiency RMCE. Genetic crossing then is used to link traits via meiotic recombination. The tightly linked trait genes can then be introgressed into other inbreds as a single locus. The relatively short, but adequate genetic distance between these transgenes also allows removal of a transgene if needed. This CTL approach was not possible previously because targeted gene insertion in crop plants was inefficient before the advent of CRISPR-Cas9 technology. Our results show that the CRISPR-Cas9 system enables robust gene targeting via homology directed repair (HDR) and can be used to establish trait gene landing sites in maize elite inbred lines. We found that transgene expression was consistent across the preselected sites and that inserted transgenes had minimal effects on neighboring endogenous gene expression. As expected, these sites can be genetically linked through traditional crossing. CTLs enable efficient and flexible production of maize hybrids with multiple transgenic traits. Insertion of SSILP at these sites had no impact on yield.

## Results

### Selection of Chromosomal Location for Complex Trait Loci

**FIGURE 1:**
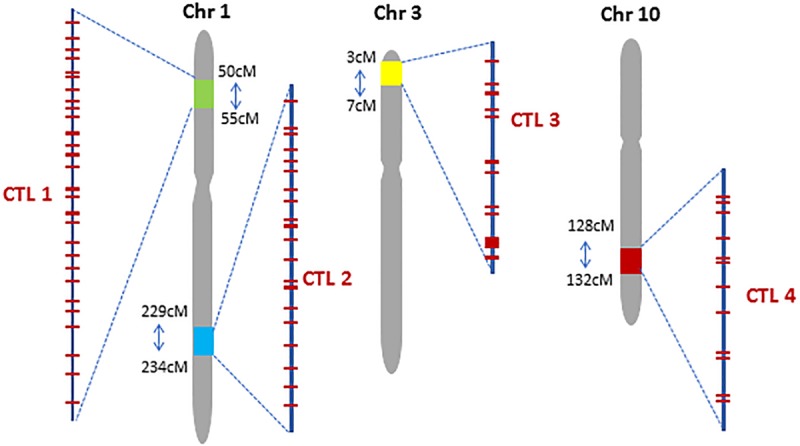
Chromosomal location of four Complex Trait Loci (CTL) in the maize genome. Red bars within each CTL represent preselected CRISPR targeting sites.

We used four criteria to decide where to locate CTLs in the maize genome: (1) regions with conserved haplotype within non-stiff stalk (NSS) and stiff stalk (SS) germplasm pools; (2) regions with low gene density that are not used in forward breeding; (3) regions with high recombination frequency to minimize the donor sequences around the CTL while introgressing the region; (4) regions harboring existing commercially valuable traits. Genomic sequences of >1,000 elite lines were scanned using a 10-cM window to identify regions of 4–5 cM to serve as CTLs. We selected four chromosomal regions to generate CTLs in maize which we refer to as CTL1, 2, 3, and 4, respectively (Figure 1). In the four CTLs, the DNA sequence was conserved among 84, 44, 34, and 55% of the SS inbreds and among 56, 72, 73, and 84% of the NSS inbreds, respectively. The regions had a gene density of 20, 21, 4 and 8 genes per cM and an average ratio of physical-to-genetic distance of 0.4, 0.6, 0.2, and 0.2 Mb/cM, respectively, as estimated based on the maize B73 reference genome sequence v2. A stacked insect-resistant and herbicide-tolerant maize event DP-004114-3 (Diehn et al., 2013) resides at 53.5 cM on chromosome 1, which is within CTL1. CTL3 is in a telomeric region of Chr 3, which can facilitate TI with a single crossover.

### Selection of CRISPR-Cas9 Target Sites Within CTLs

CRISPR-Cas9 was used to introduce trait genes into preselected sites within each CTL. To minimize regulatory concerns and product development costs, CRISPR-Cas9 target sites (CTS) were selected based on the following criteria: (1) the target site is at least 2 kb away from any known gene; (2) the CTS DNA sequence is unique in the genome and conserved among the targeted inbred lines; (3) the genomic sequences of 200–500 bp flanking the CTS are unique in the genome; and (4) spacing of the CTSs within a CTL would accommodate genetic crossing to recombine traits. A total of 30, 21, 13, and 12 CTSs were selected for CTL1, CTL2, CTL3, and CTL4, respectively (Figure 1, Table 1, and Supplementary Table S1). These sites spanned 4.18 cM (2.5 million base pairs, Mbp) at CTL1, 4.28 cM (3.2 Mbp) at CTL2, 2.35 cM (0.6 Mbp) at CTL3, and 3.04 cM (0.7 Mbp) at CTL4. Most of the target sites were 0.1–3 cM apart, suitable for both genetic stacking and subsequent segregation as a single locus.

**TABLE 1:** CRISPR-Cas9 mediated insertion of SSILP in preselected sites at CTL1 in PH184C.

**CRISPR target site**	**Genetic location (cM)**	**Number of shoot regenerated**	**Number of shoot with target site modified**	**Target site modification frequency**	**Number of shoot positive HDR1**	**Number of shoot positive HDR2**	**Number of shoot positive 2 × HDR**	**2 × HDR frequency**
TS49	50.87	214	198	93%	2	4	3	1.4%
TS50	50.95	263	218	83%	4	8	8	3%
TS51	51.06	300	280	93%	4	5	11	3.7%
TS41	51.27	356	220	62%	7	7	11	3.1%
TS71^∧^	51.32	979	871	89%	25	13	59	6%
TS72	51.33	309	287	93%	7	4	7	2.3%
TS81	51.45	220	170	77%	3	6	6	2.7%
TS73	51.48	252	191	76%	1	4	7	2.8%
TS14	51.54	293	277	95%	4	3	2	0.7%
TS74	51.61	161	129	80%	1	4	1	0.6%
TS75*^∧^	51.68	899	716	80%	4	6	15	2.6%
TS84*	51.68	366	273	75%	6	7	6	1.6%
TS76	51.69	264	198	75%	3	5	18	6.8%
TS77^∧^	51.72	666	502	75%	8	7	14	2.1%
TS78	51.75	329	188	57%	1	1	7	2.1%
TS19	51.95	217	17	8%	0	1	0	0%
TS85	51.95	217	168	77%	1	3	1	0.5%
TS86	52.54	216	183	85%	1	1	6	3.7%
TS8	52.56	217	205	95%	2	2	9	4.1%
TS43	52.8	179	140	78%	1	5	3	1.7%
TS11	53.15	177	174	98%	2	6	7	4%
TS47	53.21	200	171	86%	1	2	7	3.5%
TS80	53.23	336	330	98%	3	1	4	1.2%
TS52	53.25	222	151	68%	0	2	0	0%
TS87	53.57	302	298	99%	6	2	12	4%
TS88	53.59	370	193	52%	3	2	1	0.3%
TS45^∧^	53.66	616	562	91%	16	10	36	5.8%
TS44	54.16	246	221	90%	0	1	9	3.7%
TS46	54.43	244	220	90%	7	0	5	2%
TS10	54.56	224	209	93%	4	4	4	1.8%

Mutation of target sites (TS) in the regenerated shoots was detected using a quantitative PCR (qPCR) assay. Insertion events with both the right and left junctions positive (2 × HDR) were identified using nested junction PCR assays. *Two target sites at the same approximate genetic location, physically 6 kb apart from each other. ^∧^Data from two rounds of transformation.

### Targeting Site-Specific Insertion Landing Pad to Preselected Sites Using CRISPR-Cas9

**FIGURE 2:**
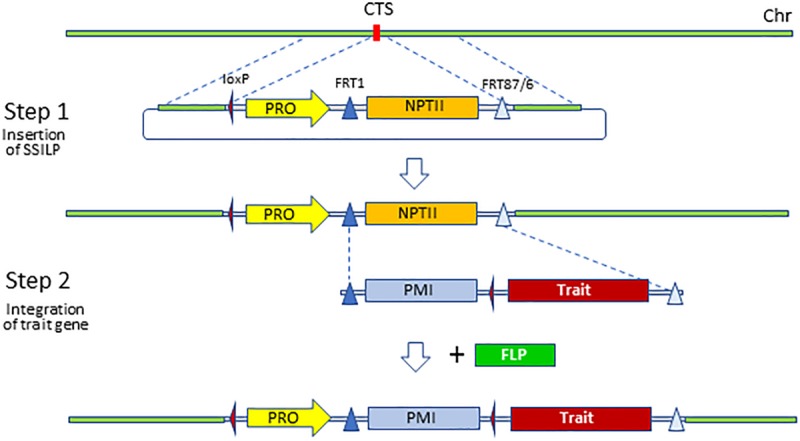
Two-step strategy to integrate trait gene to preselected sites. Site-specific insertion landing pad (SSILP) was inserted to a CRISPR-Cas9 target site (CTS) via homology-directed repair. Lines containing the SSILP can be retransformed with a trait gene which is integrated to the SSILP via RMCE. The PRO in SSILP serves as promoter trap for the selection marker in the trait gene vector. The selectable marker phosphomannose isomerase (PMI) can be removed via CRE-mediated recombination if desired. FLP, flippase recombinase; FRT, flippase recognition target; loxP, side triangle, locus of X-over P1 site; PRO, promoter; NTPII, neomycin phosphotransferase II; Chr, chromosome.

Although trait genes can be inserted directly at the selected CTS via HDR, efficiencies for CRISPR-Cas9-enabled gene insertion are low (Svitashev et al., 2015; Endo et al., 2016; Shi et al., 2017; Danilo et al., 2018; Hummel et al., 2018). Because many constructs need to be screened and tested in the early stages of product development, it is not practical to directly insert trait gene cassettes on a large scale using HDR at present. Therefore, we adopted a two-step strategy for trait gene insertion (Figure 2). First, a SSILP about 3 kb in length was inserted into target sites using CRISPR-Cas9. Trait gene cassettes were then integrated into the SSILP via RMCE in a second transformation of the characterized SSILP transgenic plants (Li et al., 2009; Anand et al., 2019). RMCE utilizes the flippase (FLP) recombinase and FLP recognition targets (FRT) to insert a gene in the target site, and has been shown to be an efficient technique for site-specific transgene insertion (Li et al., 2009; Ow, 2011; Srivastava and Thomson, 2016; Anand et al., 2019).

**FIGURE 3:**
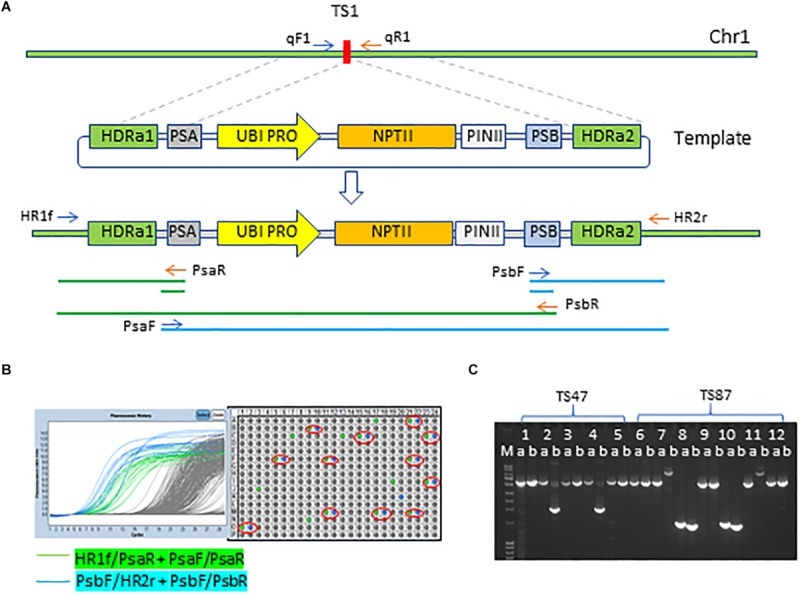
Insertion of SSI landing pad to CRISPR-Cas9 target site. **(A)** Insertion of SSI landing pad (SSILP) into CRISPR-Cas9 target site 1 (TS1) on chromosome 1 (Chr 1). UBI PRO, maize UBIQUITIN1 promoter; NPTII, neomycin phosphotransferase II; PINII, potato proteinase inhibitor II terminator; HDRa, homology-directed repair arm; PSA and PSB, unique sequences to facilitate high-throughput screening for insertion events. HR1f, HR2r, PsaR, PsaF, PsbF, and PsbR are PCR primers and arrows indicate the direction of primers. **(B)** Screening of T0 plants for insertion events with PCR and qPCR. The plants positive for both junctions are indicated in red circles. **(C)** Image of agarose gel showing overlapping long PCR products for two SSILP insertion sites (TS47 and TS87). Lane a, PCR products amplified with primers HR1f and PsbR; Lane b, PCR products amplified with primers PsaF and HR2r; Lane M, molecular-weight size markers. PCR products of the sample 1, 3, 5, 6, 9, and 12 have expected size.

To insert SSILPs at preselected sites, immature embryos were co-bombarded with four DNA plasmids (Supplementary Figure S1) containing: repair template, *Streptococcus pyogenes* Cas9, guide RNA, and the maize morphogenic genes *Baby boom* (*Bbm*) and *WUSCHEL2* (*Wus2*) (Svitashev et al., 2015; Lowe et al., 2016). The DNA repair template consisted of the SSILP flanked by two DNA sequences of approximately 400-bp homologous to the genomic sequences immediately adjacent to the CTS (Figure 3A). Two unique sequences, PSA and PSB, flanking the SSILP also were included to facilitate high-throughput PCR screening (Figure 3A). The same SSILP sequence was used for all target sites, but the homologous arms varied to match the genomic sequences bordering each CTS. Approximately, 1,000 immature embryos per CTS were used for genotypes PH184C and HC69 while 500 immature embryos were used for PHH5G because it has a higher transformation frequency.

We used junction PCR assays to detect SSILP insertion in T0 plants regenerated from embryogenic calli. In this assay, PCR amplification of the target region was coupled with nested quantitative PCR (qPCR) to detect SSILPs (Figures 3A,B). Plants positive for both 5′ and 3′ junctions (hereafter referred to as 2 × HDR events) were further analyzed with overlapping long PCR (Figures 3A,C). In PH184C, events with SSILP insertion were identified for 28 out of 30 sites at CTL1, 19 out of 20 sites at CTL2, 13 out of 13 sites at CTL3, and 10 out of 12 sites at CTL4 (Table 1 and Supplementary Table S1). Among the 28 sites at CTL1, the insertion frequency varied from 0.3 to 7.1% (Table 1). The site TS13 at CTL4 had the highest insertion frequency with 18% of the events positive for both junctions (Supplementary Table S1). A selected subset of CTSs in CTL1 also were targeted for SSILP insertion in the inbreds PHH5G and HC69. Positive events were identified for all sites and the insertion frequency was generally similar to that seen in PH184C. While most of the 2 × HDR T0 plants had mono-allelic insertion at the CTS, we found three events with bi-allelic insertion of SSILP at the CTS.

We identified some 2 × HDR T0 plants that were free of the helper genes (Cas9, gRNA, *Bbm*, and *Wus2*), indicating that transient helper gene expression during transformation can be sufficient to enable homologous gene targeting. However, most of the 2 × HDR plants contained one or more copies of the helper genes. To remove any helper DNA sequences and repair template that might have randomly inserted into the genome, the 2 × HDR T0 plants were crossed to recurrent parent (RP, the wild type of the same inbred line as initially transformed) to produce T1 seeds, and T1 plants were crossed to RP again to generate T2 seeds. PCR assays were used to detect the presence of helper genes. Out of the 89 CTSs with 2 × HDR events, we obtained helper gene-free T1 plants for 67 sites (Table 2). The integrity of inserted SSILPs at each CTS and the absence of the helper genes and other plasmid DNA fragments in the genome were further verified using Southern-by-Sequencing (SbS) analysis (Zastrow-Hayes et al., 2015); perfect SSILPs at 57 CTS out of the 67 sites were confirmed by SbS (Table 2).

**TABLE 2:** Insertion of SSILP in preselected target sites at four complex trait loci (CTL) and recovery of clean plants free of genome-editing helper genes.

**CTL**	**Genotype**	**Sites targeted**	**Sites 2 × HDR**	**Sites 2 × HDR T1 seed**	**Sites 2 × HDR and null helpers (qPCR)**	**Sites 2 × HDR perfect and null helpers (SbS)**
1	PH184C	36	34	24	19	15
1	HC69	6	6	6	6	3
1	PHH5G	24	24	18	17	17
2	PH184C	20	20	15	11	10
2	HC69	11	10	9	6	5
3	PH184C	13	13	9	4	3
4	PH184C	12	10	8	4	4

*Thirty unique sites within CTL1 in the inbred line PH184C were targeted using donor vectors containing FRT1/87. Among those 30 sites, six also were targeted using the donor vector carrying FRT1/6. A subset of 6 and 24 out of the 30 sites were targeted in HC69 and PHH5G, respectively. For CTL2, SSILP was inserted into 20 unique sites in PH184C. Only 11 out of the 21 sites were targeted in HC69. 2 × HDR, both junctions PCR positive; SbS, Southern-by-Sequencing.*

### Transgene Expression at CTL Sites

**FIGURE 4:**
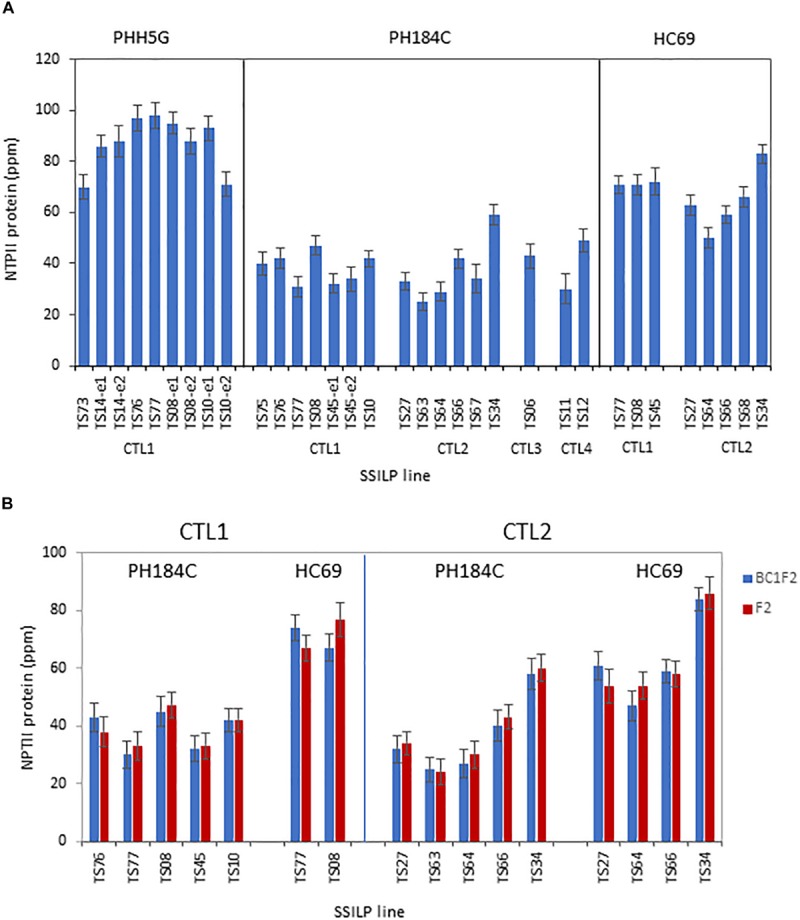
Protein expression of the *NPTII* gene targeted to preselected sites at CTLs. **(A)** NPTII protein content in leaves of 3-week-old plants was measured using ELISA. The plants contain one copy of SSILP and were selected based on PCR genotyping results. One to two independent insertion events per target site were analyzed. Error bars, SE; *N* = 8–32. A total of 33 lines were measured in the study. **(B)** NPTII protein content in leaves of 3-week-old BC1F2 and F2 PH184C or HC69 plants was quantified using ELISA. The plants contain one copy of SSILP and were selected based on PCR genotyping results. One event per target site was analyzed. Error bars, SE; *N* = 7–17. A total of 16 lines were measured in the study.

For useful transgenic trait development, genomic sites must be able to support transgene expression. To assess the effect of insertion site on transgene expression, protein expression levels of the Neomycin phosphotransferase II (*NPTII*) gene in SSILPs were measured. In the PH184C lines, the NPTII protein in leaves averaged 38 ppm with relatively low variation among sites, either within or across CTLs (Figure 4). Some, or perhaps all the observed variation is not due to insertion site effect since similar expression variation was observed among independent events at a single site, such as TS10-e1 and TS10-e2 at 54.56 cM in PHH5G. There are a small number of sites where gene expression was different from the norm. For example, the SSILP at TS34 at CTL2 in two different genetic backgrounds expressed NPTII approximately 33% higher than any of the other 15 sites tested (Figure 4). Genetic background was found to have a greater influence on the NPTII expression levels than genomic location. For the sites at CTL1, the average NPTII protein content was 37, 69 and 83 ppm in PH184C, HC69, and PHH5G, respectively. Similarly, the CTL2 sites in HC69 had a higher NPTII expression than that in PH184C (Figure 4). Since all sites tested support high level transgene expression and the position effect is smaller than the genotype background effect, we believe that these SSILPs are suitable for product development.

### Effects of Site-Specific Insertion Landing Pad Insertion on Expression of Neighboring Endogenous Genes

**FIGURE 5:**
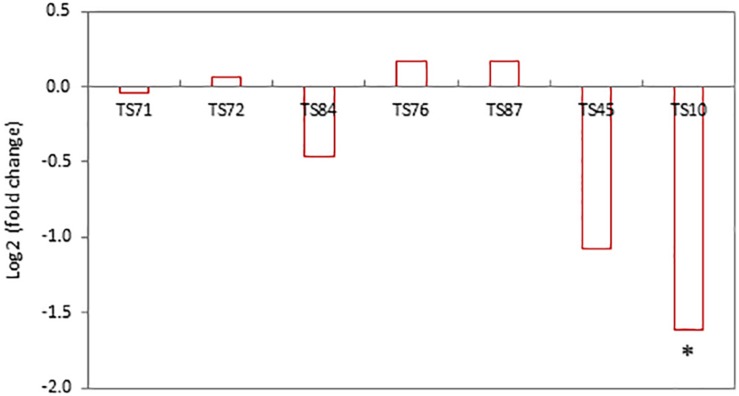
Expression of Zm0001d027859 in PH184C lines with SSILP inserted in the CTL1 region compared to that in the lines with insertion in CTL2. RNA-sequencing was performed to determine gene expression in the leaf tissues of 2-week-old seedlings. The transcript levels of the endogenous genes in the vicinity of CTL1 target sites in seven lines were compared to the CTL2 control. Contrasts are presented as log2 (fold change). Asterisk indicates false discovery-corrected *P* < 0.05.

One concern related to the random- or targeted-insertion of a transgene in the plant genome is that the insert may affect the expression of neighboring endogenous genes. As an attempt to minimize interactions of SSILPs with nearby genes, we selected target sites that were at least 2 kb away from endogenous genes. To test if this distance is adequate, RNA sequencing was used to analyze nine PH184C lines, including seven lines with SSILPs inserted at CTL1 and two lines with insertions at CTL2. Because the two CTLs are 175 cM apart, the CTL2 SSILP lines were used as comparator to determine the effect of SSILP insertion on nearby genes within the CTL1 region. In the vicinity of the CTL1 target sites from 49.45 to 55.47 cM, there were 83 endogenous maize genes, of which 69 expressed in the leaf tissues of PH184C (Supplementary Table S2). None of these genes showed significant differential expression [absolute value of log2 (fold change) <1; false discovery-corrected *P* > 0.05] in pairwise comparisons between a CTL1 insertion line and the control, except for one gene, Zm00001d027859. The transcript level of this gene was reduced 67% in the line TS10 [log2 (fold change) = −1.61 and false discovery-corrected *P* = 0.049; Figure 5]. However, this gene was not differentially expressed in the other six CTL1 insertion lines relative to the CTL2 comparator. Zm00001d027859 is located at the genetic position 54.58 cM, approximately 5 kb downstream of the SSILP insertion site at 54.56 cM in the line TS10. This spatial proximity is likely responsible for the observed reduction in expression. Overall, these results indicate that the SSILPs generated in this study have little impact on the expression of neighboring endogenous genes.

### Integration of Trait Genes to Site-Specific Insertion Landing Pads

Next, we evaluated whether the SSILP lines generated in this study were competent for RMCE. Eleven CTL1 SSILP lines in PH184C were used for trait gene insertion via FLP/FRT-mediated RMCE (Table 3, Figure 2). A total of 326 donor constructs were tested in seven SSILP lines using particle bombardment. Putative T0 RMCE plants were generated for most of the constructs at the seven sites with an average of 4% recovery (T0 plants/embryos used). Among T0 plants, ∼45% were quality events that had a single copy of the trait gene integrated into the SSILP (Table 3). We also tested RMCE using *Agrobacterium*-mediated transformation; the putative T0 RMCE recovery rate ranged from 5.9 to 9.3% in four SSILP lines tested (Table 3). These results indicate that all SSILP lines tested are capable of high-efficiency RMCE.

**TABLE 3:** Integration of trait gene to SSILP at CTL1 in PH184C.

**SSILP location**	**FRT**	**Delivery method**	**Number of constructs**	**Transformation frequency (percent)**	RMCE* ratio (percent)	**Quality event rate (percent)**
TS50	1/87	PB	326	3.9	38	1.5
TS71	1/87	PB	326	3.8	50	1.4
TS84	1/87	PB	326	3.2	42	1.1
TS76	1/87	PB	326	4.4	53	1.7
TS8	1/87	PB	326	4.3	48	1.8
TS43	1/87	PB	326	4.5	43	1.6
TS45	1/87	PB	326	3.7	45	1.5
TS77	1/6	Agro	74	7.8	58	4.6
TS8	1/6	Agro	74	9.3	52	4.9
TS45	1/6	Agro	74	5.9	55	3.2
TS10	1/6	Agro	74	8.4	53	4.4

Data are averages of 326 PB constructs or 74 Agro constructs. The frequency of transformation is the number of regenerated T0 plants for each construct divided by the number of embryos used in transformation. The RMCE ratio is the number of RMCE events divided by the number of T0 plants analyzed with PCR. The quality event rate is the number of RMCE events divided by the number of embryos used in transformation. SSILP, site-specific insertion landing pad; FRT, flippase (FLP)/FLP-recombinase targets; PB, particle bombardment; Agro, *Agrobacterium*-mediated transformation. *RMCE events are characterized by (1) presence of single intact copy of the donor genes (*PMI*, *PAT*, trait gene); (2) absence of the marker gene *NPTII*; (3) presence of FRT1 and FRT87 or FRT6 junctions; and (4) absence of unintended DNA sequence insertion including that derived from vector backbone, *Bbm*, *Wus2*, and *FLP* gene.

### Trait Stacking via Genetic Recombination

To determine if trait genes inserted at SSILPs in two different lines can be linked on the same chromosome through genetic recombination, PH184C inbred lines containing a SSILP at various sites in CTL1 were tested. Previously, we had generated a transgenic PH184C line named M14, which carries the phosphomannose isomerase (*PMI*) gene at 51.54 cM on Chr 1 within CTL1. This line was crossed with homozygous plants from 15 SSILPs (Table 4). The F1 progeny from each cross were backcrossed to WT inbred PH184C. BC1 seeds were assayed using qPCR to determine the presence or absence of the *PMI* gene and the *NPTII* gene at the SSILP. Most progeny will have either *PMI* or *NPTII*. However, a small number of progeny are expected to have chromosomal crossover between the two genes, and will have both *PMI* and *NPTII* or neither of them. The genetic distance between the two genes was calculated based on the recombination frequency (i.e., 1% recombinants is equal to 1 cM). For 14 out of 15 crosses, the observed genetic distance between M14 (*PMI*) and SSILP (*NPTII*) closely matched the predicted genetic distance based on the B73 reference genome (Table 4). For the cross of M14 with SSILP-TS72, no recombinants were recovered from 1,400 BC1 seeds analyzed. The SSILP-TS72 is located 0.17 cM from the PMI insertion site, the closest SSILP among the 15 sites. A larger BC1 population may need to be screened to identify recombinants for this SSILP. These results demonstrate that the genes integrated into SSILPs in CTL1 can be stacked by genetic crossing.

**TABLE 4:** Genetic stacking of *NPTII* in SSILPs with *PMI* at the chromosomal location 51.54 cM within CTL1.

**Target site**	**Predicated genetic position (cM)**	**Predicated distance between M14 and SSILP (cM)**	**Number of BC1 seeds**	**Number of recombinants**	**Observed genetic distance (CM)**
TS50	50.95	0.55	2688	15	0.56
TS41	51.27	0.23	2016	5	0.25
TS71	51.32	0.18	2079	3	0.14
TS72	51.33	0.17	1481	0	0.00
TS84	51.68	0.18	1153	4	0.35
TS75	51.68	0.18	1932	7	0.36
TS76	51.69	0.19	1587	2	0.13
TS78	51.75	0.25	1001	4	0.40
TS86	52.54	1.04	1291	9	0.70
TS8	52.56	1.06	1748	15	0.86
TS43	52.80	1.3	3578	36	1.01
TS11	53.15	1.65	1721	22	1.28
TS87	53.57	2.07	2261	53	2.34
TS45	53.66	2.16	1509	36	2.39
TS10	54.56	3.06	1913	55	2.88

*Maize PH184C plants homozygous for the *NPTII* gene, which has been inserted in preselected target sites at CTL1 as part of the site-specific insertion landing pad (SSILP), were crossed with the M14 line which contains the herbicide resistance gene *PMI* at the location 51.54 cM on Chromosome 1. The resulting F1 progenies were crossed with the wild-type PH184C, producing BC1 seeds. Genotyping of BC1 seeds was performed with qPCR assays. Recombinants, seeds were positive for both *NPTII* and *PMI* and null for both *NPTII* and *PMI*.*

### Presence of Site-Specific Insertion Landing Pad Has No Impact on Grain Yield

**FIGURE 6:**
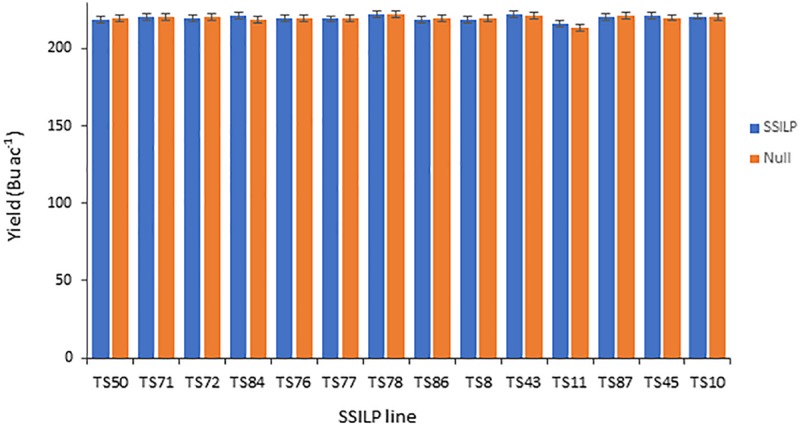
Grain yield of maize hybrids containing SSILP and corresponding nulls. Yield trials were carried out in the United States corn-belt in 2017. Each bar represents data from three hybrids at 12 locations. All analyses were implemented using ASReml with output of the model presented as best linear unbiased prediction (BLUP). The yield of the SSILP hybrids were not significantly different from their corresponding nulls (*P* > 0.05, two-tailed test).

To be useful for transgenic product development, CTL insertion sites must be agronomically neutral. To evaluate the impact of SSILP insertion at CRISPR target sites on plant productivity, we ran a multi-location hybrid field trial in the United States. In this trial, lines were evaluated for grain yield and grain moisture. Plant and ear height, and growing degree units required for pollen shed and silking were also measured. SSILP lines at 14 CRISPR target sites in the PH184C background were used as pollen donors (BC1F3 generation) to cross with three elite tester lines to make hybrids hemizygous for the CTL transgenes. As controls for each SSILP line, a null segregant at the BC1F2 generation was used to produce BC1F3 plants as pollen donors for hybrid seed production. Three hybrids for the 14 SSILP lines and corresponding nulls were planted at 12 locations. The yields were similar among the 14 SSILP lines and no difference was observed between the hybrid with SSILP and it’s corresponding null segregants (Figure 6). The trials demonstrated no yield impact attributable to SSILP insertion in all 14 lines. Like yield, other non-yield traits measured did not show a significant difference between the SSILP hybrids and nulls.

## Discussion

To construct complex trait loci, we have used CRISPR-Cas9 to generate many independent maize target lines, each containing a single SSILP in one of four preselected genomic regions. DNA sequence analyses confirmed precise insertion of SSILPs via HDR. The *NPTII* gene in the SSILP functioned properly as evidenced by resistance to G418, and we observed minimal variation in its expression level among different insertion sites within a genotype but significant difference between genotypes. Expression of endogenous genes neighboring SSILP insertions was largely unaffected. Trait genes were integrated into the SSILP with high efficiency via RMCE. By crossing the SSILP lines with a pre-established insertion event within the CTL1, we demonstrated that SSILPs could be linked through genetic recombination.

Complex trait loci have several desirable characteristics. First, they enable high-quality assembly of products with multiple traits by effectively creating a single genetic locus for TI. Second, given the ease of crossing and screening recombinants, traits can be added and removed as needed. Third, because trait genes are inserted in well characterized locations, many of the costs associated with testing novel construct-site combinations are eliminated.

This CTL approach is different from previously reported transgene stacking using RMCE (Ow, 2011; Nandy et al., 2015; Srivastava and Thomson, 2016; Chen and Ow, 2017). CRISPR-Cas-enabled gene targeting allows precise positioning of trait genes within a small, preselected region on a chromosome. In contrast, recombinase-mediated gene insertion is molecular stacking, and relies on a randomly inserted site in the genome. The resulting molecular linkage between two transgenes can’t be broken easily in genetic crossing and the closeness of the stacked transgenes raises concerns about expression interactions. Although SSILP/recombinase were used to insert trait genes in a two-step process, they are not essential components for a CTL. A trait gene could be directly inserted into a preselected site within a CTL using CRISPR-Cas9. We used a two-step process because it enables more efficient generation of transgenic lines. By using a pre-established SSILP at preselected sites, many constructs can be easily evaluated with a very small number of events since those insertion sites are well-characterized.

A few insights into plant gene targeting via HDR and gene expression were generated as part of our CTL construction and characterization. This is the largest example to date using CRISPR-Cas9 to promote homology-directed insertion in maize. We recovered targeted insertion events in 93% of tested sites (69 out of 74). As expected, no insertion was obtained without efficient DNA cleavage, as shown in TS19 at CTL1 (Table 1), TS77 at CTL2 and TS15 at CTL4 (Supplementary Table S1). However, a high mutation frequency did not always result in a high frequency of insertion, for example TS52 at CTL1, TS62-HC69 at CTL2, and TS5 at CTL4 (Table 1 and Supplementary Table S1). Other studies have concluded that several factors, including chromatin structure, DNA sequence of target sites and homology arms can influence the efficiency of CRISPR-Cas9 and HDR (Kuscu et al., 2014; Wu et al., 2014; Liu et al., 2016). We did observe variation across different sites, but overall the CRISPR-Cas system is robust, and we could obtain HDR at most sites. Although CRISPR-Cas9 from *S. pyogenes* (SpyCas9) was used successfully to insert a SSILP at numerous preselected sites in this study, other CRISPR systems like Cas12a (Cpf1), Cas9 orthologs from *Streptococcus thermophilus* (SthCas9) and *Staphylococcus aureus* (SaCas9) could also be used for construction of CTLs in maize and other crops (Steinert et al., 2015; Begemann et al., 2017; Tang et al., 2017), or combined with SpyCas9 to increase target density.

To obtain a usable SSILP, HDR must take place at the target site. In this study, we were able to obtain 2 × HDR events with high frequency. We also found events with HDR occurring only at one end of the SSILP insert while the other end likely was repaired through NHEJ because the junction PCR was negative. These 1 × HDR events often had truncations, insertion of other plasmid fragments or rearrangement of the template DNA. We selected events for 2 × HDR and against 1 × HDR using junction PCR, long PCR and sequencing, and the insert sequence integrity of the selected 2 × HDR events was verified by SbS analysis. The sites were mostly transformed once. Why some sites were more efficient than other sites SSILP insertion requires further study.

Four plasmids were co-bombarded to insert a SSILP to CRISPR target sites. We adopted this strategy, instead of using an all-in-one plasmid, because vectors containing one or two gene expression cassettes are easy to construct and it allows changes in components and plasmid ratio in transformation. Although using individual plasmids may increase the possibility of random plasmid insertion at different locations in the genome, this work and previous studies have shown co-bombarded plasmids tend to insert at the same location in the genome, especially when the morphogenic genes are used in transformation (Gao et al., 2020). We obtained helper-gene free plants for the majority of the SSILP lines generated. The random insertion of co-bombarded plasmids was not a significant limiting factor in populating CTLs with SSILP.

Selected SSILP lines were tested for reception of trait genes using many constructs. RMCE frequency with *Agrobacterium*-mediated transformation was higher than that in bombardment. These results likely are due to different FRT sites used in the site-specific integration. In *Agrobacterium*-mediated transformation, the SSILP and donor vectors had the FRT1/6 pair while the FRT1/87 pair was used in the SSILP and donor in bombardment. It has been reported that the FRT1/6 had a lower excision rate than FRT1/87 when the FLP recombinase was present (Anand et al., 2019). There is a 1 nt difference between FRT1 and FRT87 in the spacer region, but FRT1 and FRT6 differ by 3 nts. FRT cross-reactivity was found to be negatively correlated with RMCE frequency, and the relationship was more pronounced in *Agrobacterium*-mediated transformation. The RMCE frequency was not significantly different between FRT1/87 and FRT1/6 when using bombardment (Li et al., 2009; Anand et al., 2019).

We found that the plants containing SSILP had normal growth and development in the greenhouse and in the field. The SSILP insertion sites were preselected at least 2 kb away from any known gene. RNA-seq analysis demonstrated that insertion of SSILP had no significant effect on expression of nearby endogenous genes. The SSILP plants were generated by back-crossing with wildtype twice. Our work on CRISPR-waxy corn product development (Gao et al., 2020) and several other studies using CRISPR-Cas9 has found very limited or no off-target cutting in plants (Tang et al., 2018; Hahn and Nekrasov, 2019; Li et al., 2019; Young et al., 2019). A few albino seedlings were found among the selfed BC1F2 plants in two events. However, this albino phenotype was not caused by SSILP insertion, was seen in null segregants as well, and is a common occurrence in maize genetics. We selected SSILP from events without abnormal plants. Occasional off-type plants also are seen in traditional genetic crosses or transgenic regenerants; we observed nothing unusual in this CRISPR-Cas9 mediated gene insertion work. Importantly, yield test showed that SSILPs were agronomically neutral when compared to null segregants.

Most sites supported similar level transgene expression within an inbred line, a result that is consistent with earlier reports (Chawla et al., 2006; Nanto et al., 2009; Betts et al., 2019). It has been proposed that transgenic event recovery is dependent upon the ability of the selectable marker or screenable marker to be expressed. Because of this, events in regions of the genome where silencing occur will not be recovered (Francis and Spiker, 2004). While it is possible such repressive locations exist in the maize genome, in this report most of the sites tested supported gene expression, suggesting that at least in the chromosome regions studied here, consistent transgene expression at preselected sites is the norm. It is noteworthy that expression at identical sites was significantly different across different genetic backgrounds, PH184C < HC69 < PHH5G. Given the ability to target a unique SSI landing pad at multiple genetically identical target sites across different maize elite inbreds, this is the first report on examining how differences in genetic background influence gene expression without the linkage-related complications associated with backcrossing.

## Materials and Methods

### Plant Materials and Growth Conditions

Three Pioneer^®^ inbred lines PH184C, HC69, and PHH5G were used in these experiments. M14 is a transgenic PH184C line generated via meganuclease mediated HDR, carrying the selectable marker gene *PMI*. SSILP lines used in transformation for trait gene integration were heterozygous plants from crossing of homozygous BC1F3 or later generations with wild type PH184C. Embryo donor plants and transgenic plants were grown in greenhouses as previously described (Shi et al., 2017).

### CRISPR-Cas9 Target Sites Selection

The genetic and physical location of CTSs on chromosomes were calculated based on marker prediction using the B73 reference genome. The DNA sequences from the chromosomal region of the four CTLs were scanned using proprietary bioinformatic tools for unique sequence regions at least 2 kb away from any native gene, then potential CTSs were identified by first locating a suitable PAM for *S. pyogenes* Cas9, NGG and then extracting the sequence between 17 and 24 bp 5′ of the PAM for use as the spacer in the sgRNA. The off-targeting cutting potential of Cas9 based on the selected CTSs was evaluated by searching the B73 reference genome and transformation inbred lines for closely matching targets using Bowtie 2 and PSI-BLAST (Altschul et al., 1997; Langmead and Salzberg, 2012). Only CTSs different from other genomic locations by at least two mismatches in the target site seed region were selected (1–10 bp 5′ of the PAM).

### Plasmid Construction and Maize Transformation

The single guide RNA gene consists of a maize U6 polymerase III promoter, a CRISPR RNA, a trans-activating CRISPR RNA and a terminator (Supplementary Figure S1). The Cas9 expression cassette contains the maize UBIQUITIN1 promoter (UBI PRO), *S. pyogenes* Cas9 endonuclease and potato protease inhibitor II terminator (PINII). The Cas9 DNA sequence was maize codon optimized and the potato ST-LS1 intron and the nuclear localization signals from the SV40 were added for appropriate expression and nuclear targeting in maize, as previously described (Svitashev et al., 2015). Constructs were assembled using chemically synthesized DNA fragments with standard DNA techniques. *NPTII* served as a transformation selection marker. To improve regeneration of plants, morphogenic regulators *Bbm* (also known as ovule development protein 2 or *ODP2*) and *Wus2* were expressed under control of the maize UBI1 promoter and In2-2 promoter, respectively (Supplementary Figure S1), and the plasmids were constructed as described previously (Lowe et al., 2016).

Embryos from PHH5G line used in SSILP insertion contained a pre-integrated T-DNA of *Bbm* and *Wus2* to enhance transformation. Biolistic-mediated transformation of maize immature embryos was performed as previously described (Svitashev et al., 2015). Briefly, gold particles, 0.6 μm in diameter, were washed with 100% ethanol and sterile distilled water. The plasmid DNA purified with QIAprep Spin Miniprep (Qiagen, Germany) and mixture of Cas9-gRNA/donor template/BBM/WUS2 = 5/5/2.5/2.5 was precipitated on the washed gold particles using a water-soluble cationic lipid TransIT-2020 (Mirus). Fifty microliters of gold particles (water solution of 10 mg/mL) and 1 μL of TransIT-2020 water solution were added to the premixed DNA, mixed gently. DNA-coated gold particles were then centrifuged at 8,000 *g* for 1 min. The pellet was rinsed with 100 μL of 100% ethanol and re-suspended by a brief sonication. Immediately after sonication, DNA-coated gold particles were loaded onto the center of a macro-carrier (10 μL of each) and allowed to air dry. Immature embryos 9–11 days after pollination were bombarded using a PDS-1000 Helium Gun (Bio-Rad) with a rupture pressure of 425 psi. Post-bombardment culture, selection, and plant regeneration were carried out as described (Svitashev et al., 2015).

To integrate a trait gene to the SSILP, plasmids used in *Agrobacterium*-mediated transformation contain six expression cassettes: the trait gene and phosphinothricin acetyltransferase (*PAT*) gene in the donor, the transformation selection marker *PMI* (promoter-less), the transformation enhancer *Bbm* and *WUS2*, and FLP recombinase for RMCE (Supplementary Figure S2). The coding sequences, promoters and terminators as well as FRT and loxP were PCR-amplified or chemically synthesized, verified by DNA sequencing and assembled in a Gateway-modified derivative of pSB11. The plasmids then were co-integrated into the super binary pSB1 vector in *Agrobacterium tumefaciens* strain LBA4404 by electroporation. *Agrobacterium*-mediated transformation of maize immature embryos was performed as described previously (Lowe et al., 2016). For biolistic-mediated transformation, individual plasmids of UBI:WUS2, UBI:BBM, and UBI:FLP were co-delivered with the donor plasmid containing *PMI* and the trait gene flanked by FRT1 and FRT87 sites to immature embryos as described above.

### DNA Extraction and Genotyping by PCR

Genomic DNA was extracted from leaves as described previously (Shi et al., 2017). A qPCR assay was used to estimate the copy number of each CTS. Shoots with no modification contain two copies of the wild-type CTS, shoots with CTS modification either due to NHEJ or SSILP insertion in one of the two homologous chromosomes has one intact copy, while modification in both chromosomes would reduce the copy number to zero. qPCR was performed using Qiagen QuantiTect Multiplex PCR Master Mix (Qiagen, Germany) primers and probe specific for each CTS (Supplementary Table S3). Junction PCR assays were used to detect SSILP insertion at each CTS. In this assay, to increase screening throughput, PCR amplification of the target region was coupled with a nested qPCR to detect SSILPs. PCR was performed using 2x Extract-N-amp PCR Ready Mix (Cat# E3004, Sigma) or 2× Phusion Flash High-fidelity PCR Master Mix (Cas#F548L, Thermo Fisher Scientific). For nested PCR used in screening SSILP insertion events, the first PCR was carried out in 5 μL of reaction mixtures for 20 cycles. Fifteen μL of the reaction mixture containing 2×TagMan Master Mix (LGC Cat# KBS-1001-001) and primers then were added, and the second PCR was performed using LightCycler 480 (Roche Life Science) for 30 cycles. Data were analyzed using the Endpoint Genotyping Software (Roche Life Science). Long PCR was performed using Extensor Master Mix (Cat# AB-0792, Thermo Fisher Scientific). HR1f and HR2r primers which varied among CTS, primers and probes are listed in Supplementary Table S3. To detect trait gene integration at the SSILP via FLP mediated RMCE, qPCR was performed (Supplementary Table S4). The qPCR for identifying recombinants from crossing of M14 and the SSILP lines was performed using Qiagen QuantiTect Multiplex PCR Master Mix (Qiagen, Germany). Primers and probes are listed in Supplementary Table S4.

### Detection of Plasmid DNA in Plants

Presence of plasmid DNA in the genome of T0 or T1 plants was determined by qPCR and SbS. QPCR was performed using Qiagen QuantiTect Multiplex PCR Master Mix (Qiagen, Germany) with primers and probes listed in Supplementary Table S4. SbS was performed as described by Zastrow-Hayes et al. (2015). A capture-probe library was created to cover the four plasmids used in transformation (Supplementary Figure S1). Illumina whole-genome sequencing libraries were constructed from DNA derived from plants. Hybridizations and sequencing were carried out as described (Zastrow-Hayes et al., 2015).

### Quantification of Neomycin Phosphotransferase II Proteins by ELISA

Plants were grown in greenhouse in 4 × 8 cell flats in a randomized complete block design. Leaf punches were taken from the third leaves of 3-week-old greenhouse grown heterozygous plants. The leaf samples were ground in 500 μL PBST with two metal beads using a Geno/Grinder at 1,650 rpm for 60 s followed by centrifugation at 4°C (3,889 *g*) for 10 min. Total protein was quantified using the Bradford Protein Assay Kit (Bio-Rad) per the manufacturer’s instructions. The NPTII protein was measured using a polyclonal antibody-based sandwich ELISA assay as previously described (Schmidt and Alarcon, 2011). The NPTII specific polyclonal antibody assay standard curve was linear from 2 to 40 ng/mL. Samples were assayed in duplicate with comparison of interpolations across varying sample dilution. Controls (negative and known low and high positive) were included on each assay plate. Results were reported as parts per million based on total protein.

### Endogenous Gene Expression Analysis by RNA Sequencing

Plants were grown in greenhouse in 4 × 8 cell flats in a randomized complete block design. Leaf samples for the SSILP lines and controls were taken from 2-week-old seedlings in four biological replicates. Each biological replicate consists of 3–4 individual plants. Total RNAs were extracted using the Qiagen RNeasy kit for total RNA isolation (Qiagen, Germantown, MD, United States). Sequencing libraries from the resulting total RNAs were prepared using the TruSeq mRNA-Seq kit according to the manufacturer’s instructions (Illumina, San Diego, CA, United States) and sequenced on the Illumina HiSeq 2500 system with Illumina TruSeq SBS version 3 reagents. Reads were trimmed based on quality scores, filtered and mapped to the PH184C reference transcripts using the aligner software Bowtie2 v2.3.4.1 (Langmead and Salzberg, 2012). The PH184C reference sequences were established using following steps: First, the PH184C CTL1 scaffold was mapped to B73 RefGen v4.59 using Minimap v2.14-r883 to determine the general location of CTL1 in the genome (Li, 2016; Jiao et al., 2017). Next, the B73 gene sequences from this region were extracted from RefGen v4.59 and mapped to the PH184C CTL1 scaffold using GMAP v2018-07-04 (Wu and Watanabe, 2005). Then the PH184C mapped gene sequences on the CTL1 scaffold, which could be slightly different from the B73 sequences, were extracted and served as the reference transcripts for RNA-seq analysis (Portwood et al., 2019).

### Statistical Analysis of RNA-Seq Data

RSEM v1.2.28 was used to estimate transcript abundance (Li and Dewey, 2011). Initial exploratory analysis of the aligned sequences showed high variability due to plant location in the greenhouse, which contributed significantly to the overall variability in the sequencing data. Spatial affects were captured using surrogate variable analysis (svaseq package in R) (Leek, 2014). Using the DESeq2 package in R, differential analysis was performed by first selecting likelihood-ratio test models to test for overall significance of the SSILP site variable (Love et al., 2014). The full model included the SSILP site information and surrogate variables found in the surrogate variable analysis, while the null model included only the surrogate variables to account for spatial variability. The Wald test then was performed for pairwise comparisons of each SSILP site against the CTL2 control. The differential expression is defined as the false discovery-corrected *P* < 0.05 and the absolute value of log2 (fold change) > 1.

### Hybrid Seed Production and Yield Test

Regenerated T0 plants containing a SSILP were backcrossed to respective wild type inbred line for two successive generations followed by selfing to develop BC1F2 seeds. Plants homozygous for the SSILP or null segregant which did not contain the SSILP were selfed to produce BC1F3 seeds. BC1F3 plants were planted in the field, and individual plants were selected for uniformity. Selected BC1F3 plants were crossed to three elite tester female lines to produce hybrid seeds for yield testing. F1 hybrid seeds were therefore either null or hemizygous for a SSILP.

Hybrid yield testing was conducted at 12 locations in the United States. The experimental design was two-row plots nested by tester and a set of SSILP lines and corresponding null segregants as controls. Fertilizer was applied, and weeds and pests were controlled according to local practices. Small plot combines were used to collect grain mass and grain moisture data. Grain yield was calculated by normalizing all entries to 15% moisture.

A mixed model framework was used to perform the multi-location data analyses (Betts et al., 2019). Analysis was implemented using ASReml (VSN International Ltd.), and the values are best linear unbiased prediction (BLUP) (Gilmour et al., 2009). The SSILP hybrids were compared to corresponding null-segregant controls, and statistical differences were determined at *P* < 0.05.

### Material Availability

Novel biological materials described in this publication may be available to the academic community and other not-for-profit institutions solely for non-commercial research purposes upon acceptance and signing of a material transfer agreement between the author’s institution and the requestor. In some cases, such materials may originally contain genetic elements described in the manuscript that were obtained from a third party(s), and the authors may not be able to provide materials including third party genetic elements to the requestor because of certain third-party contractual restrictions placed on the author’s institution. In such cases, the requester will be required to obtain such materials directly from the third party. The authors and authors’ institution do not make any express or implied permission(s) to the requester to make, use, sell, offer for sale, or import third party proprietary materials. Obtaining any such permission(s) will be the sole responsibility of the requestor. To protect Corteva Agriscience’s proprietary germplasm, such germplasm will not be made available except at the discretion of Corteva Agriscience and then only in accordance with all applicable governmental regulations.

## Data Availability Statement

All data supporting the findings of this study are available in the article/Supplementary Material. RNA sequencing data are available in the SRA database in the BioProject PRJNA612862. Plasmids accession number are MT221180, MT221181, MT221182, and MT221179 correspond plasmid 1, 2, 3, and 4 on Supplementary Figure S1.

## Author Contributions

HG designed, coordinated experiments. JY and HG selected CTS. JM selected CTL regions. JM and HG conducted stacks. MY, MS, and BL performed molecular analysis on SSILPs. LW constructed DNA vectors. DP, GS, SJ, LF, WM, MZ, SW performed plant transformation. JF and KS conducted sampling and plant care. NC designed RNA-seq experiment. CS and HG led gene expression studies. XS performed statistical design and analysis for gene expression. JS led the trait gene insertion experiments. JM conducted field trial. SB, AC, NC, and HG designed SSILP structure. HG and NC wrote the manuscript. JM, JY, XS, JS, SB, and AC edited the manuscript.

## Conflict of Interest

All authors were employees of Corteva Agriscience at the time of their contributions to any experiments works. Patents related to this work have been filed.
