# Supplementary material for: Complex Trait Loci in Maize Enabled by CRISPR-Cas9 Mediated Gene Insertion
Source: Front Plant Sci. 2020 May 5;11:535. doi: 10.3389/fpls.2020.00535 (PMC7214728; doi:10.3389/fpls.2020.00535)
Supplement: Supplementary file 1 [file Table_1.pdf]

Table S1. Targeted insertion of SSILP at preselected sites in PH184C and HC69.

Mutation of target sites (TS) in the regenerated shoots was detected using a quantitative PCR (qPCR) assay. Insertion events with the left (HDR1) or right (HDR2) junction or both junctions positive (2xHDR) were identified using junction PCR assays. Carets denote data from two rounds of transformation.

CTL	Genotype	CRISPR target site	Genetic location (cM)	Number of shoot regenerated	Number of shoot with target site modified	Target site modification frequency	Number of shoot positive HDR1 only	Number of shoot positive HDR2 only	Number of shoot positive 2xHDR	2xHDR frequency
2	PH184C	TS71	229.03	333	263	79%	9	7	14	3.6%
		TS72	229.52	512	333	89%	6	11	12	2.3%
		TS73	229.53	470	299	80%	14	8	40	8.5%
		TS74	229.65	455	332	88%	8	12	18	4.0%
		TS75	229.66	447	n/a	n/a	6	3	8	1.8%
		TS76	229.77	457	321	85%	15	12	22	4.8%
		TS77	229.83	470	6	1%	0	0	0	0.0%
		TS62^	230.55	589	299	95%	10	5	14	2.4%
		TS27	230.83	236	227	96%	0	3	4	1.7%
		TS63	230.85	246	225	91%	1	4	15	6.1%
		TS64	230.92	334	281	84%	3	3	17	5.1%
		TS79	231.18	564	235	63%	3	4	7	1.2%
		TS80	231.37	564	282	75%	4	2	3	0.5%
		TS30^	231.38	778	351	93%	19	22	44	5.8%
		TS65	231.47	447	403	90%	6	4	13	2.9%
		TS66	231.69	408	365	89%	2	1	2	0.5%
		TS67	232.1	242	234	97%	2	3	10	4.1%
		TS68^	232.2	640	275	84%	9	20	16	2.5%
		TS34	232.82	160	138	86%	0	5	2	1.3%
		TS69	233.31	309	306	99%	2	1	2	0.6%
2	HC69	TS62	230.55	628	584	93%	1	1	0	0.0%
		TS27	230.83	597	522	87%	5	2	7	1.2%
		TS63	230.85	644	553	86%	9	3	12	1.9%
		TS64	230.92	602	485	81%	4	4	7	1.2%
		TS30	231.38	349	281	81%	1	0	1	0.3%
		TS65	231.47	556	543	98%	1	4	1	0.2%
		TS66	231.69	533	413	77%	5	4	6	1.1%
		TS67	232.1	305	290	95%	2	0	4	1.3%
		TS68	232.2	454	400	88%	7	2	3	0.7%
		TS34	232.82	793	722	91%	1	0	15	1.9%
		TS69	233.31	533	405	76%	1	2	4	0.8%
3	PH184C	TS3	4.63	604	530	88%	22	15	21	4.0%
		TS6	5.55	491	425	87%	15	7	13	3.1%
		TS7	5.56	345	195	57%	3	3	24	12.3%
		TS8	5.69	603	116	19%	4	1	3	2.6%
		TS9	5.7	384	247	64%	5	4	18	7.3%
		TS10	5.93	453	145	32%	0	2	4	2.8%
		TS11	5.94	598	317	53%	2	2	6	1.9%
		TS13	6.12	514	336	65%	5	9	14	4.2%
		TS14	6.32	361	288	80%	2	3	8	2.8%
		TS16	6.9	501	414	83%	11	12	24	5.8%
		TS17	6.91	431	380	88%	15	13	30	7.9%
		TS18	6.92	376	192	51%	0	12	5	2.6%
		TS19	6.98	594	466	78%	7	12	10	2.1%
4	PH184C	TS2	128.13	149	146	98%	1	2	3	2%
		TS3	128.14	264	252	95%	6	9	15	6%
		TS4	128.16	174	159	91%	3	7	6	4%
		TS5	129.23	324	294	91%	1	0	0	0%
		TS7	129.54	265	226	85%	7	5	5	2%
		TS8	129.55	600	556	93%	9	1	4	1%
		TS9	130.16	484	445	92%	16	24	38	9%
		TS11	130.42	681	644	95%	31	37	67	10%
		TS12	130.94	491	415	85%	19	13	36	9%
		TS13	130.95	554	387	70%	25	19	70	18%
		TS15	131.16	561	29	5%	0	0	0	0%
		TS16	131.17	453	400	88%	22	9	32	8%
