# Supplementary material for: Complex Trait Loci in Maize Enabled by CRISPR-Cas9 Mediated Gene Insertion
Source: Front Plant Sci. 2020 May 5;11:535. doi: 10.3389/fpls.2020.00535 (PMC7214728; doi:10.3389/fpls.2020.00535)
Supplement: Supplementary file 4 [file Table_4.pdf]

Supplementary Table S4. List of PCR primers and probes.

Primers and probes used in detection of helper-genes in SSILP insertion events, recombinase-mediated cassette exchange (RMCE) events in trait gene integration at SSILP, and recombinants in *NPTII* and *PMI* stacking.

PCR/qPCR analysis	PCR for	Primer name	Sequence (5' to 3')
Helper genes in SSILP event detection			
	ADH reference	ADH-F	CAAGTCGCGGTTTTCAATCA
		ADH -R	TGAAGGTGGAAGTCCCAACAA
		ADH- probe	VIC-TGGGAAGCCTATCTACCAC
	Cas9	Cas9-F	CAGAATGAAAAGCTCTACCTCTACTACCT
		Cas9-R	TGGTCGACGTCGTAGTCCGA
		Cas9-probe	FAM-TCCTGGTCCACGTACAT
	gRNA	gRNA-F	CTAATCACAAGAGTGGAGCGTACCTT
		gRNA-R	AGCCTTATTTTAACTTGCTATTTCTAGCTCT
		gRNA-probe	FAM-CCGAGCCGCAAGCA
	BbM	ODP2-F	CGGCGATGTCTGCTTCAA
		ODP2-R	AAGCTCTGATCCCCTCATGCT
		ODP2-probe	FAM-ATCCCCCAAGATTG
	Wus2	Wus-F	CTCTGTCCGGTGTCAGTAGCAAT
		Wus-R	TGCCTCCTCCCGCTCC
		Wus-probe	FAM-ACCGCCGCCCGCA
RMCE event screening			
	FRT1 junciton	ubi_F	CTCACCCTGTTGTTTGGTGTTACTT
		pmi_R	TTGCACTGAGTTAATGAGTTTTTGC
		FRT probe	TCCTATTCCGAAGTTCCTAT
	FRT87/6 junction	pse_F	GGCTAGTGGTCACTTAGGGCTTTAA
		psb_R	AACCTACGAAGTCATCGGAATCA
		FRT probe	TCCTATTCCGAAGTTCCTAT
Recombinants in stack progeny screening			
	PMI at M14	pmi_f	CGCCGGAGATATCGTTTCA
		pmi_r	CCTCTCCGAGCAGAGTCGATT
		pmi probe	CTCAATCACATCACGCA
	NPTII at SSILP	npt2_f	CGACCACCAAGCGAAACAT
		npt2_r	CGACAAGACCGGCTTCCAT
		npt2 probe	CATCGAGCGAGCACGT
