# Supplementary material for: Complex Trait Loci in Maize Enabled by CRISPR-Cas9 Mediated Gene Insertion
Source: Front Plant Sci. 2020 May 5;11:535. doi: 10.3389/fpls.2020.00535 (PMC7214728; doi:10.3389/fpls.2020.00535)
Supplement: Supplementary file 5 [file Data_Sheet_1.pdf]

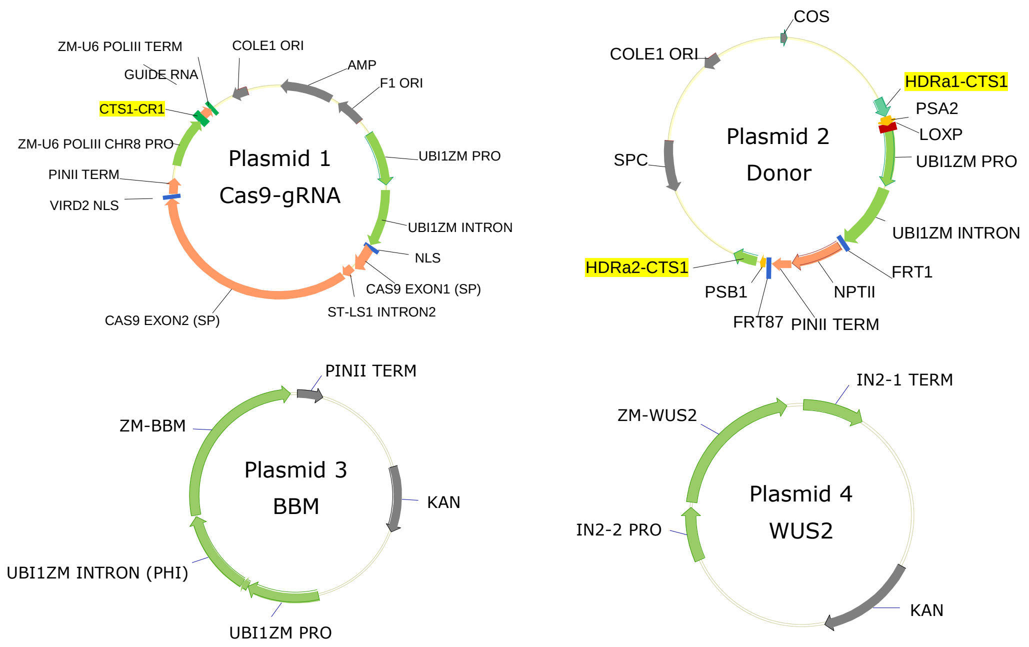

Figure S1. Plasmids used in SSILP insertion bombardment transformation

Plasmids used in CRISPR-Cas9 mediated insertion of SSILP in preselected sites. Yellow highlighted parts varies corresponding to different sites.

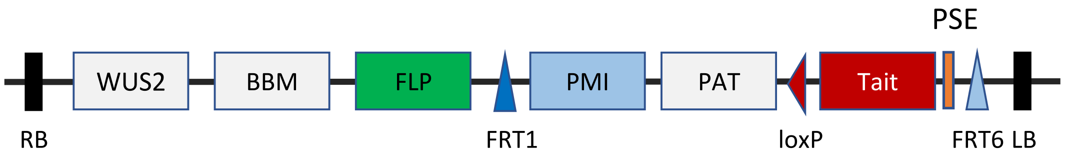

Figure S2. Schematic drawing illustrating donor vectors for trait gene integration to SSILPs via recombinase-mediated cassette exchange (RMCE).

Only the T-DNA region is shown. *WUS2* was under control of the rice Actin promoter. The expression of *BBM* and *FLP* was driven by maize UBI promoter. *PMI* was promoterless, facilitating selection of insertion in SSILPs. The *PAT* expression cassette had rice ACTIN promoter. The trait gene varied in each construct, as well as promoter and terminator. FRT, FLP recognition target; loxP, side triangle, locus of X-over P1 site. PSE, unique sequence facilitates high-throughput screening for RMCE events.
